# Accessing Different Protein Conformer Ensembles with Tunable Capillary Vibrating Sharp-Edge Spray Ionization

**DOI:** 10.1021/acs.jpcb.4c04842

**Published:** 2025-01-29

**Authors:** Daud Sharif, Vikum K. Dewasurendra, Mst Nigar Sultana, Sultan Mahmud, Chandrima Banerjee, Mohammad Rahman, Peng Li, David E. Clemmer, Matthew B. Johnson, Stephen J. Valentine

**Affiliations:** 1 Department of Chemistry, West Virginia University, Morgantown, West Virginia 26506, United States; 2 Department of Physics, West Virginia University, Morgantown, West Virginia 26506, United States; 3 Department of Chemistry, Indiana University Bloomington, Bloomington, Indiana 47405, United States

## Abstract

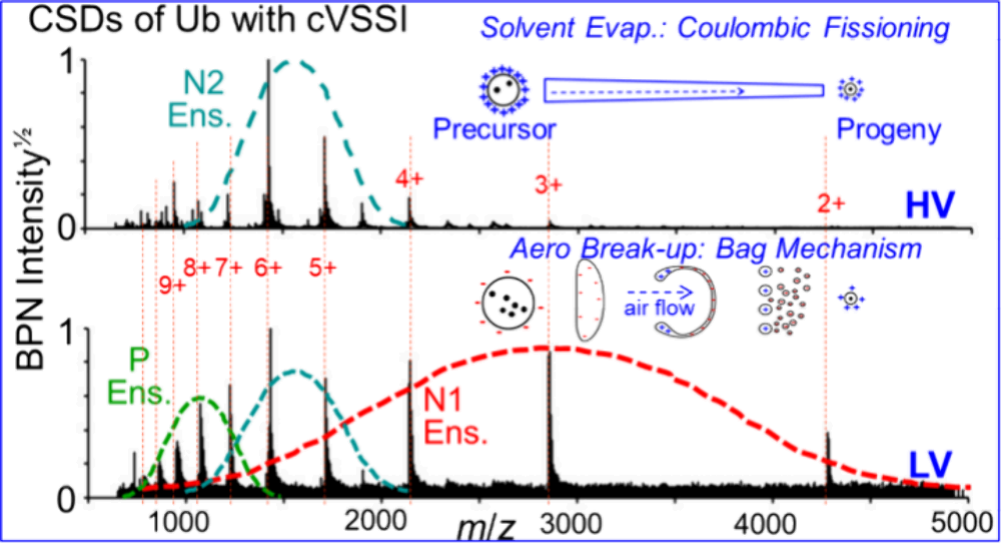

Capillary vibrating sharp-edge spray ionization (cVSSI) has been used to control the droplet charging of nebulized microdroplets and monitor effects on protein ion conformation makeup as determined by mass spectrometry (MS). Here it is observed that the application of voltage results in noticeable differences to the charge state distributions (CSDs) of ubiquitin ions. The data can be described most generally in three distinct voltage regions: Under low-voltage conditions (<+200 V, LV regime), low charge states (2+ to 4+ ions) dominate the mass spectra. For midvoltage conditions (+200 to +600 V, MV regime), higher charge states (7+ to 12+ ions) are observed. For high-voltage conditions (>+600 V, HV regime), the “nano-electrospray ionization (nESI)-type distribution” is achieved in which the 6+ and 5+ species are observed as the dominant ions. Analysis of these results suggests that different pathways to progeny nanodroplet production result in the observed ions. For the LV regime, aerodynamic breakup leads to low charge progeny droplets that are selective for the native solution conformation ensemble of ubiquitin (minus multimeric species). In the MV regime, the large droplets persist for longer periods of time, leading to droplet heating and a shift in the conformation ensemble to partially unfolded species. In the HV regime, droplets access progeny nanodroplets faster, leading to native conformation ensemble sampling as indicated by the observed nESI-type CSD. The notable observation of limited multimer formation and adduct ion formation in the LV regime is hypothesized to result from droplet aero breakup resulting in protein and charge carrier partitioning in sampled progeny droplets. The tunable droplet charging afforded by cVSSI presents opportunities to study the effects of the droplet charge, droplet size, and mass spectrometer inlet temperature on the conformer ensemble sampled by the mass spectrometer. Additionally, the approach may provide a tool for rapid comparison of protein stabilities.

## Introduction

In recent years, the field of native mass spectrometry (MS) has grown at a rapid rate. A Web of Science topical search indicates a 30% annual growth rate from 2005 to 2020. From the early observation of multimeric protein ion species^1^ in electrospray ionization (ESI)^2^ MS data sets, native MS has broadly expanded to include all manner of biopolymer assemblies as well as the interactions between many different biomolecules and their ligands.^3^ Over the past decade a number of new technologies have been developed/advanced for usage in the field of native MS. These have been recently reviewed and reported in separate studies and include tools such as charge detection MS (CDMS)^4^ ion mobility spectrometry (IMS),^5,6^ various ion activation/fragmentation methods,^7^ the use of solution chemistry,^6,8^ the use of gas-phase ion chemistry,^9^ as well as new data analysis/workflows/computational tools.^10^ One noteworthy area experiencing rapid change is the development of high-resolution MS instrumentation and techniques.^3^

An instrumentation component of great importance for carrying out successful native MS analysis is the ionization source. Currently, nanoelectrospray ionization (nESI)^11^ is the typical choice of researchers to conduct these experiments. nESI offers advantages in sensitivity that are unmatched by traditional ESI sources utilizing gas-assisted nebulization and operating at higher flow rates. Recent seminal work has demonstrated that the use of smaller (submicron) nESI emitter tips permits the use of more native-like solutions (*i.e*., higher salt concentrations) for protein structure characterization.^12^ Voltage-free ionization sources such as surface acoustic wave nebulization (SAWN),^13^ mechanospray ionization (MoSI),^14^ and capillary vibrating sharp-edge spray ionization (cVSSI)^15^ have also been shown to be even gentler than traditional spray-based sources in the production of ions for aqueous samples. Indeed, cVSSI was shown to preserve the structures of fragile proteins such as leptin and thioredoxin, whereas approaches that utilized voltage resulted in partial protein unfolding.

Useful applications of native MS measurements include the determination of biopolymer structures and relative stabilities.^16^ In such studies, two general experimental types emerge. These are embodied in experiments employing pre- and postionization characterization techniques. Examples of the latter include the use of ion activation/fragmentation strategies such as collision-induced dissociation (CID),^17^ surface-induced dissociation (SID),^18^ infrared multiphoton dissociation (IRMPD),^19^ and UV photodissociation (UVPD).^20^ Another example of the development of postionization tools is the use of extended time scale tandem IMS (IMS-IMS and IMS^n^)^21,22^ experiments to determine the relative stabilities of native-like ions.^23^ The information rich nature of tandem IMS has led to the development of new modular instrumentation providing increased capabilities for studying protein and protein complex stabilities using native MS.^24^ One recent study combined novel computational methods with IMS-IMS to study the stabilities of newly formed gas-phase ubiquitin ions and encountered a rugged energy landscape with kinetic traps.^25^ Additionally, the unique combination of ion activation with tandem IMS to monitor structural transformations represented a key development for studying protein ion stabilities.^26^ Application of this approach has become known as collision-induced unfolding (CIU),^27^ which is finding increased usage in the study of native-like ion structure stability.^28^ Another recent study demonstrated CIU with top-down native MS/MS for characterizing protein–DNA binding dynamics.^29^

Preionization techniques that allow the probing of structural stabilities have also proliferated over the past decade. One information-rich strategy utilizes the production of ions from native MS solutions with variable-temperature ESI.^30^ An interesting variant of solution denaturation to ascertain protein stabilities involves the heating of the nanodroplet plume from an ESI source using a CO_2_ laser;^31^ this approach has even been used to construct protein denaturation curves as a function of laser power and has been used to distinguish coexisting structures of myoglobin.^32^ A more recent approach for examining biopolymer stabilities in charged microdroplets involves the use of in-droplet hydrogen–deuterium exchange (HDX).^33^ For large biopolymers, experiments have shown the ability to observe coexisting protein structures,^34^ distinguish relative stabilities of G-quadruplex DNA structures,^35^ and reveal the rapid α-helix making/breaking of an unstructured peptide using HDX.^36^

This work introduces a new approach for controlled droplet charging to study the effects on protein conformer sampling by MS. Here, cVSSI with variable emitter tip bias voltage is used to alter the average charge of nebulized droplets and the resulting mass spectra are examined for changes in charge state distribution (CSD) as well as the types and forms of adduct ions produced. cVSSI with applied emitter tip voltage is unique in that droplet charging is accomplished without Taylor-cone formation making it possible to monitor the effect of differently charged droplets (inlet transfer tube temperature and droplet size are effectively constant) on the preservation of protein structure into the gas phase environment. This approach is highly tunable and reveals gradual and distinct CSD transitions with voltage. Overall, it is observed that different protein solution conformer ensembles are sampled under 1) field-free (0 V) and low voltage (LV), 2) midrange voltage (MV), and 3) high voltage (HV) conditions. The effects observed for ubiquitin are discussed considering three separate pathways to progeny nanodroplet production that ultimately select for the observed conformer ensemble. In this work, the observations for LV are similar to those for field-free cVSSI and those for HV are similar to field-enabled cVSSI in ref (34).

Over the years, a number of unique nebulization methodologies have been developed to generate droplets for MS analysis. These include sonic spray ionization^37^ which utilizes a high velocity coaxial gas flow to produce the droplets as well as the use of a vibrating orifice aerosol generator where a piezoelectric transducer attached to the orifice nebulizes a continuous-flow stream.^38,39^ To date, only a few mechanical nebulization approaches have employed an additional electric field for MS experiments. An early approach used a glass emitter cemented in a cylindrical piezoelectric element that periodically constricted to deliver a pulse of droplets in the presence of a high field.^40^ Separate experiments used array of micromachined ultrasonic electrospray (AMUSE) technology in which a variable DC voltage was superimposed on RF voltage applied to a piezoelectric transducer.^41^ The DC voltage could be varied between 0 and 500 V. More recently, surface acoustic wave nebulization (SAWN) has been used to introduce analyte droplets into the presence of a DC discharge effected by application of high voltage to a metal pin.^42^ These approaches demonstrated utility for the study of relatively low molecular weight compounds. For example, the latter work generated ions via an atmospheric pressure chemical ionization (APCI)-like mechanism and decreased ion suppression. As mentioned above, for larger protein molecules, SAWN,^13^ MoSI,^14^ and cVSSI^15^ have been used to generate ions under zero or very low field conditions and have been shown to produce very low charge states of proteins from buffered aqueous systems suggesting very gentle ionization pathways. Notably, the unique MoSI study has demonstrated that a bimodal distribution of very low charge states (along with multimer ions) could be accessed suggesting that this approach may allow the preservation of structure not observed by ESI.^14^ The cVSSI approach described here adds to these new ionization methodologies and allows for tuning of the droplet charge using a wide range of applied voltage. This tuning capability may be exploited in the future to balance enhancement of biopolymer ionization while retaining solution structure.

## Materials and Methods

### Reagents and Chemicals

High-performance liquid chromatography (HPLC) grade water from Fisher Chemical (Fair Lawn, NJ), ammonium acetate (AmAc) from Honeywell (Muskegon, MI) and ubiquitin from bovine erythrocytes (≥98%) from Sigma-Aldrich (St, Louis, MO) were used to make protein stock solution. The stock solution of ubiquitin was prepared by dissolving 1 mg of lyophilized powder protein in 1 mL of 50 mM AmAc. The solution was diluted to a final concentration of 0.2 mg·mL^–1^ for direct infusion experiments. All chemical reagents and solvents were used without further purification.

### cVSSI Device Fabrication

cVSSI devices were constructed as previously described.^15,34,43^ First, the basic cVSSI devices were fabricated (without attached capillary emitter) by affixing a standard 4.6-kHz piezoelectric actuator (Murata Electronics, Smyrna, GA) to a 1”x3” microscope cover glass slide (VWR, Radnor, PA) using 5 min epoxy (Devcon). Next, ∼10 cm lengths of standard fused-silica capillary (360 μm OD × 100 μm ID, Polymicro Technologies) were cut. The outer polyimide coating of the capillary was burned using a butane lighter and the surface was cleaned using a wet wipe before placing inside a P-2000 micropipette puller (Sutter Instruments, Novato, CA). The puller pulled each capillary into two emitter tips of roughly equal length having an orifice of ∼15 μm. The pulled emitter tips were examined using an Olympus IX73 microscope and imaging software (cellSens, Olympus Corporation, Tokyo, Japan) was used to measure the orifice diameter. A single capillary emitter tip was then attached to the glass slide in a manner such that the narrow opening protrudes from the distal edge and is at a ∼60° angle relative to the edge of the slide.

**Figure 1:**
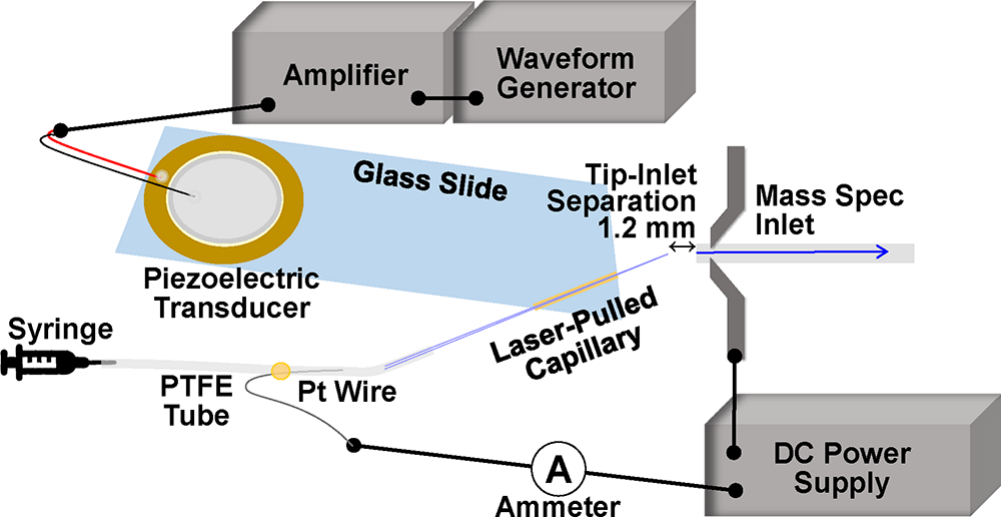
Schematic of cVSSI methodology used in these studies. Device hardware, peripheral accessories and electronics are labeled.

To deliver the analyte solution to the cVSSI-emitter tip, a 14-gauge polytetrafluoroethylene (PTFE) tubing was used. The PTFE tubing had a poly hub needle inserted and glued to one end in a manner such that the hub can accommodate the slip fit of a 1-ml plastic syringe (BD, Franklin Lakes, NJ). The other end of the PTFE tube is pushed over the blunt end of the emitter tip. To incorporate an external voltage, the PTFE tube was pierced on the device side ∼5 cm from the union of the device and the tube with an extra fine syringe needle and a Pt wire was threaded through the hole and epoxied in place. Copper tape was attached to the free end of the platinum wire to allow secure grasping by an alligator clip and a DC voltage was applied to the clip. Figure 1 shows a schematic of the cVSSI setup used for this work.

To conduct experiments, the waveform generator (Figure 1) supplied a ∼400 mV_pp_ RF voltage to the amplifier (OPA541 by Taidacent) that supplied the piezoelectric transducer with ∼10 V_pp_ RF at ∼95 kHz. Protein sample was infused through the PTFE tubing with a syringe pump (Legato 180, kd Scientific) at a flow rate of ∼2 μL·min^–1^. The tip-inlet distance was measured to be 1.2 mm. Activating the piezoelectric transducer resulted in nebulization of the infused liquid. The DC voltage applied to the Pt wire (Figure 1) was scanned from 0 V to +1000 to 0 V using a step increment of ±20 V. Notably, employing this high flow rate across this voltage range requires the use of mechanical nebulization. That is, a stable Taylor cone is not produced for these aqueous samples using DC voltage alone. Additionally, an advantage of this approach is that the nebulization produces droplets of similar size across the entire voltage range allowing the separate study of the effect of initial droplet charging (see Discussion section below).

### MS Instrumentation and Settings

All experiments were conducted on an orbitrap mass spectrometer (QExactive Hybrid Quadrupole/Orbitrap, Thermo Fisher Scientific, San Jose, CA). The data was recorded in the positive ion mode with a scan range of 650 to 5,000 mass-to-charge (*m*/*z*). The resolution was set at 70,000 with an automatic gain control (AGC) target of 5E6 and 500 ms injection time. The ion transfer tube inlet temperature was set at 275 °C. A miniature thermocouple was used to measure the air temperature directly in front of the inlet capillary. This was determined to be ∼35 °C at a distance of 1 mm. Thus, it is not expected that heating of the liquid at the capillary emitter or the large initial droplets under ambient conditions occur. This is further supported by the preservation of native structures under field-free cVSSI and field-enabled (+1000 V) cVSSI conditions (see Discussion section below and ref (15).). The default heated-ESI source was replaced with home-built housing for the cVSSI device to reduce the effect of air currents so as to ensure minimum plume spreading.

### Data Analysis

While experimenting and acquiring data using Thermo Tune software, video recordings of the software interface and plume were collected to keep track of the voltage at different data acquisition time points. MS data (.RAW) were exported as Excel files using a Python script [ref (44).]. This was performed for all MS spectra (linear interpolation to achieve evenly spaced *m*/*z*) and was necessary for signal averaging, heat map generation, etc. This was done because the *m*/*z* bins in the.RAW files are not evenly spaced, nor the same for each spectrum (ragged matrix). Start times for each spectrum were not evenly spaced as well due to AGC usage. The start times were not interpolated. The spectrum numbers and voltages were synchronized by using the timing from a video recording. Plots of charge state and adduct ion abundance with voltage were obtained by averaging the five spectra after each voltage change for the appropriate *m*/*z* range. These values were normalized using the total summed ion intensity for each voltage.

## Results and Discussion

### Experimental Data Set

In recent experiments, the use of voltage-free and field-enabled cVSSI was investigated for its ability to preserve the structures of gas-phase protein ions.^45^ Under voltage-free conditions, for all proteins studied, very low CSDs were observed as demonstrated by other field-free approaches.^13,14^ With the application of high voltage (+1,000 V), CSDs representing those similar to native MS conditions using nESI were observed for most proteins studied.^15^ However, two proteins exhibited multimodal CSDs with the application of the voltage. This was also observed for nESI and heated ESI (HESI) experiments for these proteins suggesting the possibility of a role for droplet charge in protein conformer ensemble sampling at the inlet region of the mass spectrometer. Based on these observations, the effect of droplet charge on the preservation of biomolecular structure became the focus of the present study.

It is instructive to first consider the role of cVSSI in conducting these studies. Because the droplets are produced by mechanical vibration at the sharp tip^46^ as opposed to Taylor-cone formation, it is possible to control the charge of the droplets over a much wider range than can be obtained by ESI techniques and/or other voltage-free techniques. Note that production of droplets by mechanical vibration occurs for both field-free cVSSI and field-enabled cVSSI (i.e., across the entire voltage range) as indicated in the Methods section. In this work, the voltage applied to the infused protein solution was scanned from 0 to +1,000 V in 20 V increments to observe changes in the types of biomolecular ions produced. This information was obtained in the form of the observed CSD as well as the adduct ion distribution.

The protein ubiquitin was selected for this study because its gas-phase ion structures and reactivities have been studied in detail.^21,31,47,48^ Briefly, ubiquitin in its native configuration is a tightly packed globular protein with a diameter of ∼2.5 nm and a mass of 8.565 kDa.^49^ The major secondary components, five β-sheets and an α-helix, altogether form a hydrophobic core. More importantly related to this study was the groundbreaking observation of multiple coexisting structures under native MS conditions as revealed by IMS experiments.^48,50^ More recent work involving a cryogenic drift tube has suggested that this conformational ensemble could contain a weakly bound dimer species.^51^ That said, generally higher-order multimers of ubiquitin are considered to result from nonspecific interactions that occur during droplet drying suggesting the observation of such species with a native conformer ensemble primarily results from the ionization process.^52,53^ The ubiquitin conformational ensemble was further characterized according to ensemble changes influenced by solvent composition as well as gas-phase structural transitions obtained from IMS-IMS studies.^53,54^ For discussion purposes, such conformational heterogeneity is here referred to as the native ensemble indicating native solution conditions. That is, because cVSSI produces relatively large water droplets (∼20 μm) and the solution is not heated at the emitter tip, it is assumed that the native ensemble is established within the microdroplets.

**Figure 2:**
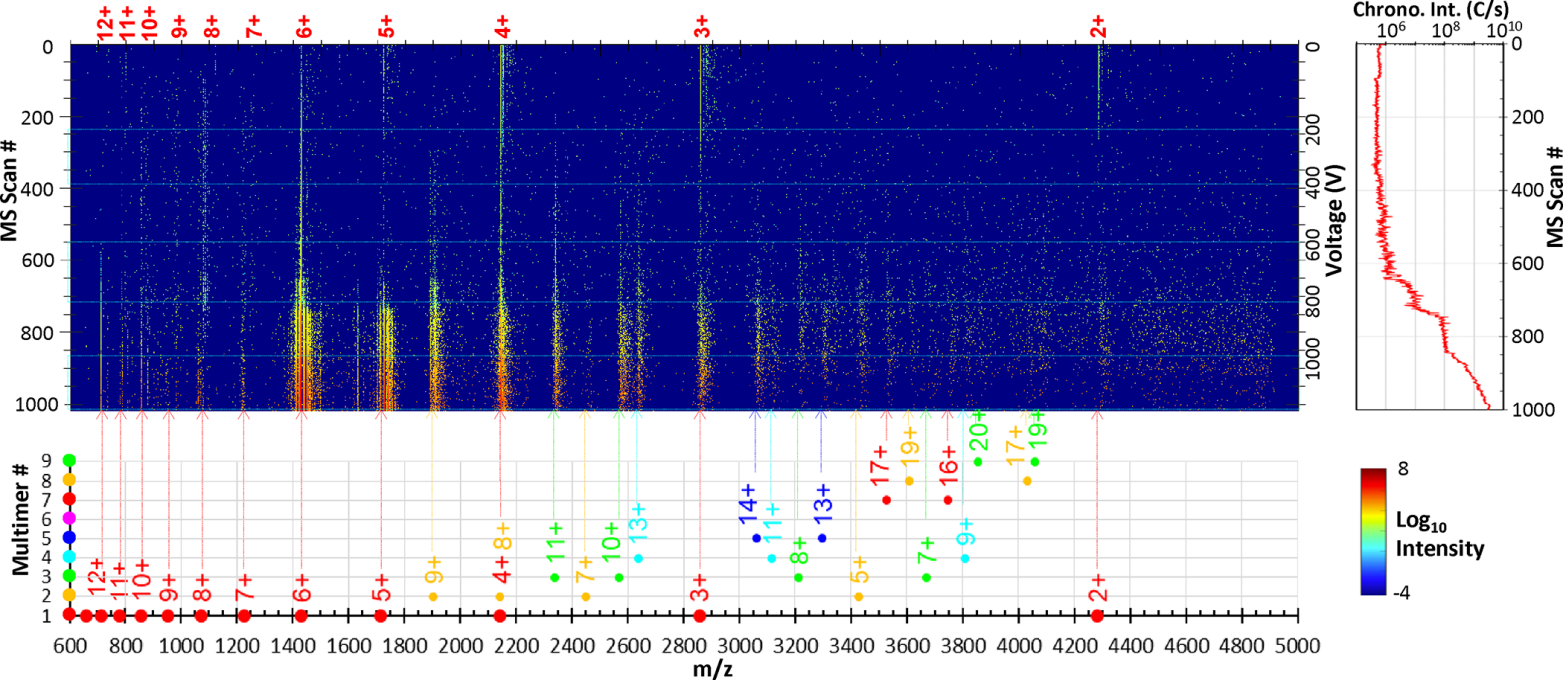
Global heat map showing all ions that are observed across the voltage range including all adduct and multimer species. The intensity is represented on a log scale. The bottom panel projects the calculated positions of different charge states of ubiquitin (including multimers). The numbers on the left represent the number of ubiquitin polypeptide units. The solid circle marks the *m*/*z* position of different charge states of ubiquitin multimer ions. The markers that are of the same color and positioned equally distant along the *y*-axis represent different charge states of the same protein or multimer ions. The panel on the right shows the total ion chronogram.

Figure 2 is a heat map that shows the mass spectral features observed as the applied DC voltage to cVSSI is scanned from 0 to +1,000 V. At low voltages the charge states 2+ to 6+ are observed in the mass spectra (horizontal axis in Figure 2) as the dominant peaks. As the voltage increases, the 2+, 3+, 4+, and 5+ ions decrease in intensity such that smaller contributions are observed at voltages > +300 V. Along with this diminished contribution to the total protein ion signal is an increase in higher charge states (7+ to 12+). By + 600 V, many features become apparent in the mass spectra. These include 2+ to 12+ charge states as well as a large number of multimeric ions (up to the [9M+20H]^20+^ ions). In this range, in decreasing order, 6+, 5+ and 4+ ions are the largest, with 6+ and 5+ ions displaying prominent adduct ion composition. The ion chronogram associated with the data set (Figure 2) shows nearly a 4 decade increase in total ion intensity as voltage is increased from 0 to +1000 V. Most of this increase occurs at voltage settings above +700 V.

### Associating CSDs and Ion Adduct Formation with Sampled Conformational Ensemble

Mass spectral CSDs are useful indicators for determining structural perturbations to the native conformers of proteins.^55^ Previously, with field-enabled cVSSI and ESI, a bimodal CSD was observed for some of the more “flexible” protein species, as determined from molecular dynamics (MD) simulations.^15^ Prior studies have also ascribed bimodal (or multimodal) CSDs to the disruption of native structure.^56^ Remarkably, the very gentle MoSI technique appears to have captured a multimodal distribution of low charge states in which different native states may be captured.^14^ As mentioned above, prior studies employing the same ubiquitin sample (nondenaturing solution) show a unimodal and narrow CSD when examined by nESI-MS or field-enabled cVSSI.^15^ In contrast, for these voltage-dependent experiments, CSD tracking reveals dynamic transitions.

**Figure 3:**
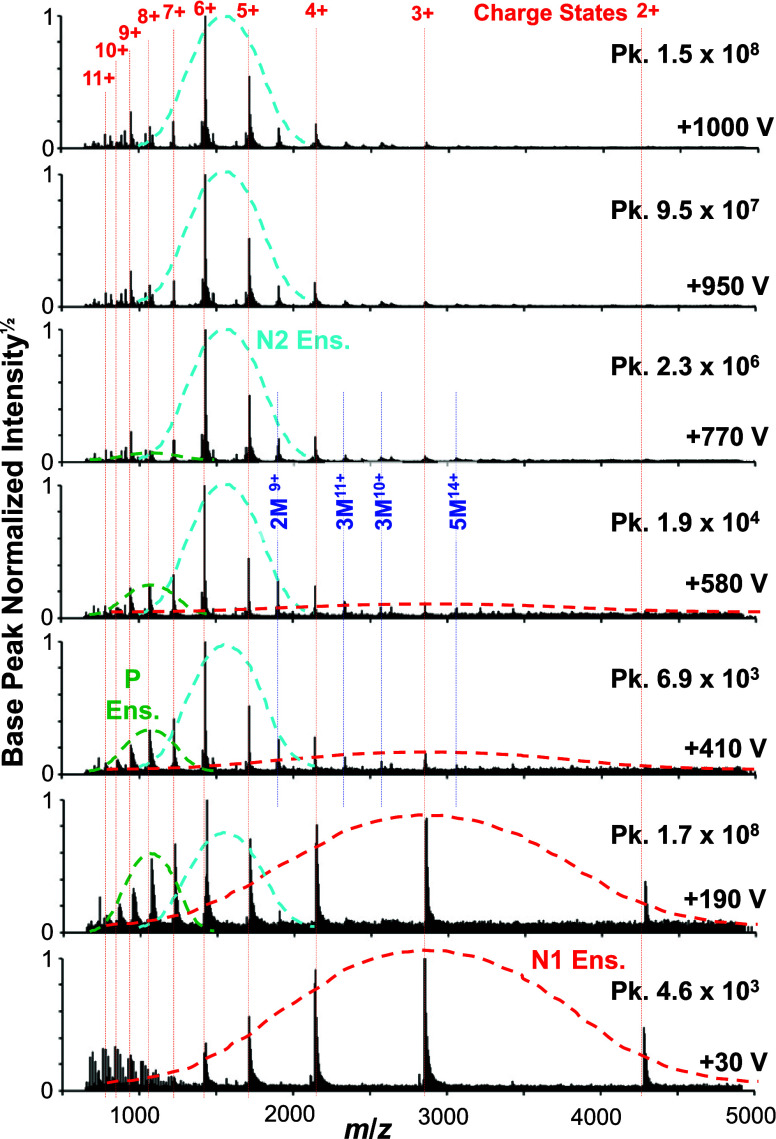
CSDs of ubiquitin resulting from ionization by cVSSI at different voltages. Different (more clearly evident) CSDs are represented by dashed lines as low-voltage N1 Ens. (red), midvoltage P Ens. (green) and nESI-type N2 Ens. (cyan). Voltages for which the respective mass spectra are obtained are indicated as an inset in each spectrum (lower right). Peak ion intensities are also provided for each spectrum (upper right). The charge states are labeled (red) at the top and lines are drawn to guide the eyes. Several prominent multimer ions are also labeled in the middle spectrum.

Figure 3 shows the comparison of the CSDs of ubiquitin from seven different voltages. Notably, at +30 V only the low charge states ions are observed and the CSD is centered at the 3+ and 4+ ions. At + 190 V the presence of the high charge state ions is evident and there is a clear bimodal distribution in the mass spectrum where that of the higher and lower charge states can be estimated to be centered at the 7+ and 8+ ions and the 3+ and 4+ ions, respectively. Here, it is also proposed that a new distribution also begins to emerge. This is suggested to be similar to the nESI-MS distribution and is centered on the 6+ and 5+ ions. As the voltage increases, the low- and high-charge-state CSDs give rise to the nESI-type CSD that dominates at +1,000 V (Figure 3).

The change in CSDs as a function of voltage may be suggestive of the sampling of different conformations within a starting ensemble and/or sampling from an ensemble resulting from structural transitions that occur in solution as protein ions do not gain charge post ionization.^57^ Therefore, assuming that the nESI-type CSD ions represents a sampling of the native conformational ensemble (N2 ensemble in Figure 3), the higher charge state CSD necessarily represents a sampling of a different conformer ensemble (i.e., partially unfolded or P ensemble in Figure 3). Here, the designation of P ensemble as opposed to unfolded (U) ensemble is used because the center of the CSD is situated near the 7+ and 8+ charge states with smaller contributions (decreasing in charge) from 9+ to 12+ ions (Figure 3). Comparatively, fully denatured (unfolded) ubiquitin presents the 11+ and 12+ species as the dominant ions and even an appreciable amount of 13+ ions.^58^ A question arises as to whether the lowest charge state CSD represents the native conformational ensemble. Here it is noted that this CSD has been labeled as a native ensemble (N1 ensemble in Figure 3) due to prior studies where only holo-myoglobin (heme containing) ions were observed at low voltage (no apo-myoglobin) in addition to a CSD consistent with the native conformational ensemble;^15^ similar assignments are encountered for voltage-free SAWN-MS^13^ and ultra low-field MoSI-MS experiments.^14^ Further evidence for these conformers within this CSD is presented below.

To further examine the relationship between different charge states at different voltage settings consider Figure S1 in the Supporting Information which shows stacked plots for 2+ to 11+ ions. Each monomer series displays ion intensity for a sequence of voltages. These series show the same features as the heat map in Figure 2 and the mass spectra shown in Figure 3 and accentuate the adduct ion distributions. For example, this includes the prominence of the 2+ to 4+ peaks at zero volts and low voltage and the prominence of the 5+ and 6+ peaks at high voltage. As will become evident below, the similarities and differences in the adduct ion peaks provide clues as to the protein conformation ensemble sampled by the mass spectrometer at the different voltages. In the following sections, the data sets are plotted in several ways to elucidate the sampled protein conformation ensemble in the different voltage ranges. In order of the discussion below, the relative intensities of the charge states and the adduct ion structure are considered. Next, these findings are related to different pathways of progeny nanodroplet formation over the different voltage ranges. Finally, the discussion centers on the process by which such pathways select for different protein conformation ensembles.

**Figure 4:**
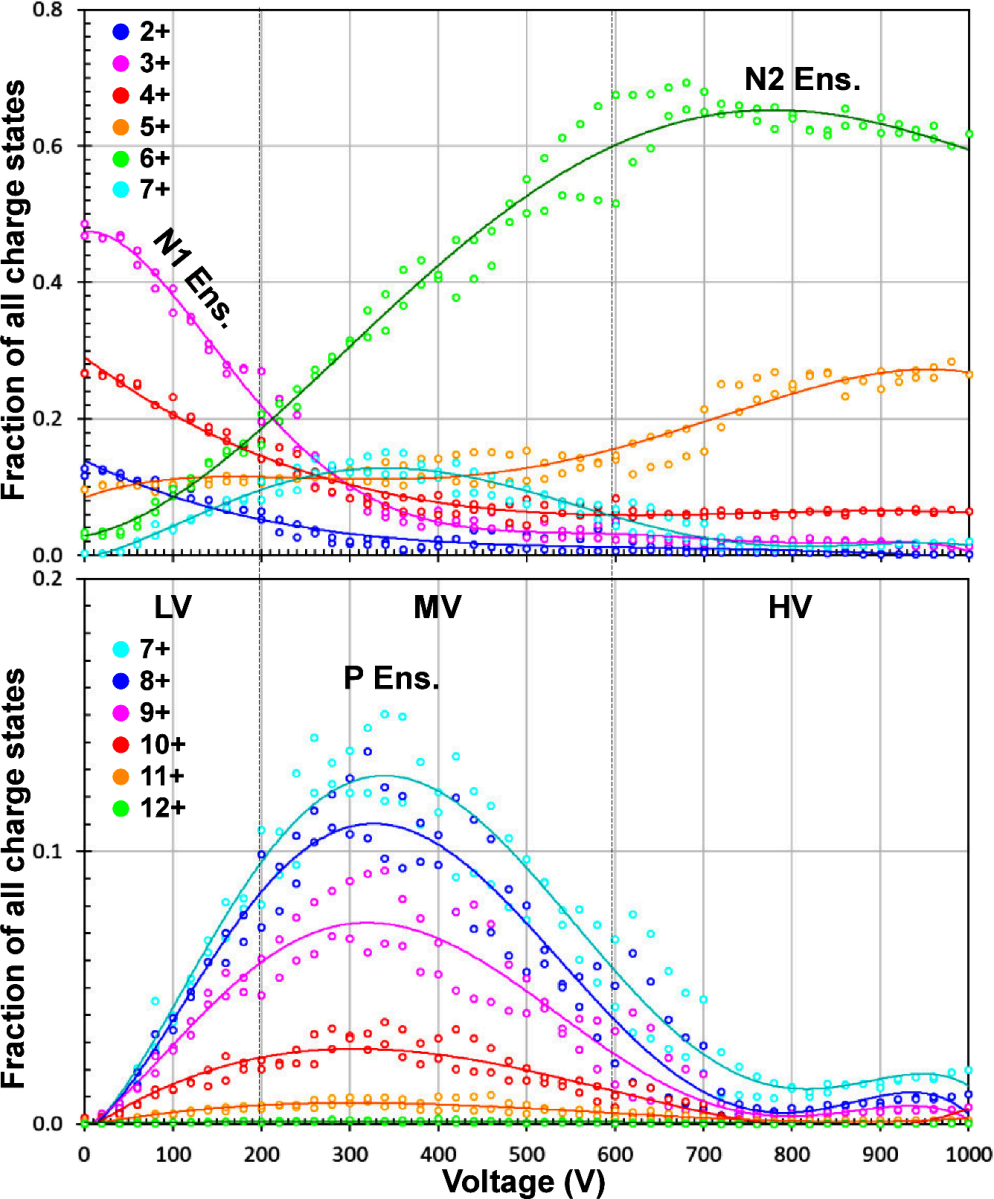
Normalized relative abundance of the different charge states (minus multimers) as a function of voltage. Starting at 0 V, for every 20 V increment, fractional proportions of the charge states (including adducts) have been measured from the observed CSDs. The charge states are split into two segments so that data visualization can be improved by minimizing overlap. The solid blue, magenta, red, yellow, green, and cyan in the top panel represent 2+, 3+, 4+, 5+, 6+, and 7+ charge states. The bottom panel shows the fractional proportions of the charge states where the solid cyan, blue, magenta, red, yellow, and green represent 7+, 8+, 9+, 10+, 11+, and 12+ charge states. Charge states representing native conformational ensembles are labeled as N1 Ens. and N2 Ens. and partially unfolded conformation ensembles are labeled as P Ens. (see text for details on these assignments).

Although Figures 2, 3, and S1 reveal the variation of ions observed in the mass spectral scans as the voltage is increased, it is instructive to consider the relative contributions of each ion in detail. Figure 4 shows the fraction of each charge state as a function of voltage. Note this plot indicates the **relative** contribution for charge state not their **absolute** ion intensities. That said, the ion intensity for the most dominant ions increases from 10^2^ to 10^8^ over this voltage range.

In Figure 4, ion fraction data are presented for spectra recorded from a stepwise voltage increase from 0 to +1,000 V and a stepwise decrease in voltage from +1,000 to 0 V. Overall, remarkable reproducibility of charge state makeup is achieved for ubiquitin for the select droplet charge (controlled by voltage). At 0 V, only low charge states are observed, which is consistent with previous cVSSI studies for globular proteins.^15^ Indeed, very low charge states are observed for even larger proteins. For ubiquitin, three broad charge state behaviors are associated with voltage. At very low voltage, 2+, 3+, 4+, and 5+ ions are observed with less 6+ and no 7+ to 12+ ions. As the voltage increases, 2+, 3+ and 4+ ions decrease monotonically reaching constant lower values by +400 V. Comparatively, the 6+ species greatly increases from 0 to +400 V and continues to increase to a broad max near +800 V and the 5+ ion contribution is roughly constant from 0 to +400 V and then increases slowly to a broad max near +900 V. The fraction of high charge state ions (7+ to 12+) all start at near zero at 0 V and build to max contributions at about +350 V and decrease symmetrically to near zero at +800 V, with the fraction of 7+ ions (highest) and the fractions of 8+ to 12+ becoming progressively smaller. In summary, the three behaviors are (see Figure 4): for low charge state ions (2+, 3+, and 4+) showing monotonic decrease with voltage from 0 to +400 V, for high charge state ions (7+ to 12+) showing symmetric features maximizing at about +350 V and near zero contributions at 0 and +800 V, and for medium charge state ions (5+ and 6+) which build from low fractions at 0 V to broad maxima at +900 and +800 V, respectively. Note the distinction between medium charge state ions and high charge state ions is abrupt with 6+ ions exhibiting maximum contribution when 7+ to 12+ ions return to near zero. Whereas the distinction between low charge state and medium charge state ions is less clear with the 5+ species present at 0 V (at 10%) vs. 6+ ions near zero at 0 V, and 4+ ions still present at high voltage (6%) vs. 2+ and 3+ ions near zero.

Based on these behaviors the 0 to +1,000 V range can be broken up into low voltage (LV) at 0 to +200 V, medium voltage (MV) at +200 to +600 V, and high voltage (HV) at +600 to +1,000 V regions. Note that the LV, MV, and HV regions correlate with prominent representation of the N1, P, and N2 ensembles, respectively, as indicated in Figure 4. Overall, the relative peak height of the dominant charge states reaches somewhat of a plateau in the range of +900 to +1,000 V. At this point, the 6+, 5+, and 4+ ions comprise about 60%, 26%, and 6% of the total protein ion abundance, respectively. It is important to note that the relative ion abundances of the different charge states formed by cVSSI at +1,000 V is similar to that expected for a native MS spectrum obtained by ESI and nESI.^59^ Although Figure 4 shows the overall change in relative ion population for the different charge states as a function of voltage, many of the charge states (*e.g*., 4+, 5+, 6+ ions) may belong to multiple/different CSD envelopes as shown in Figure 3 across this voltage range suggesting differences in the pathways leading to their production and ultimately the sampled conformational ensemble. This is discussed in greater detail below.

### Examining Adduct Ion Behavior in Greater Detail

Historically the observation (or lack thereof) of different protein ad,duct ions has been used to support given ionization mechanisms. For example, it was suggested that a greater amount of adduct ion species indicate condensation of protein and cations associated with complete droplet drying^60^ described by the charged-residue model (CRM)^61^ of ion production. Conversely, fewer adduct ion species^62^ indicate protein ion formation via the ion evaporation model (IEM).^63^ Although here, the different ionization mechanisms, including combinations thereof,^64^ are not invoked to describe the different CSDs presented in Figure 3 (and represented by intensity in Figure 4), it is instructive to compare adduct ion species for the different charge states as a function of voltage in these cVSSI studies to suggest different conformational ensemble sampling. For the comparison of different adduct ion peaks, it is useful to first visualize the ions that make up such mass spectral features obtained via cVSSI. Figure S2 shows the adduct ion distribution obtained for 6+ ubiquitin ions using +1000 V. Using binomial distribution (^13^C/^12^C) fitting, this distribution can be represented as the attachment of varying numbers and kinds of cationic species such as NH_4_^+^ and Na^+^. It is here suggested that the adduct ion distribution should therefore reflect differences in late-stage droplet composition.

With a basic description of the potential types of adduct ions formed for the ubiquitin sample, it is possible to make comparisons of the adduct ion series across different charge states and voltages. Figure S1 shows zoomed-in mass spectral regions encompassing the adduct ions for the different charge state ions. Notably, the adduct ion formation behavior of ions comprising the assigned CSDs and conformer ensembles (Figures 3 and 4) is somewhat similar across the voltage range studied. For example, Figure S2 shows that for ions comprising the low charge state CSD (N1 ensemble), at LV (0 to +200 V), very similar adduct ions are formed where the protonated (i.e., [M+nH]^n+^) species are the most intense features and up to 5 or 6 adduct ion types are observed. At slightly higher voltages, such similarities decrease. A possible contributing factor is the overlap observed between the low charge state CSD (N1 ensemble) and the nESI-type CSD (Figures 3 and 4), where the 4+ and 5+ adduct ion species begin to more resemble those of the 6+ ions. For the ions comprising the high charge state CSD (7+ to 12+ ions), unique adduct ion distributions are observed across the MV region. Thereafter at HV (>+600 V), it is difficult to distinguish the adduct ions from the high charge state CSD and those that overlap with the nESI-like CSD (particularly for the 7+ and 8+ ions).

**Figure 5:**
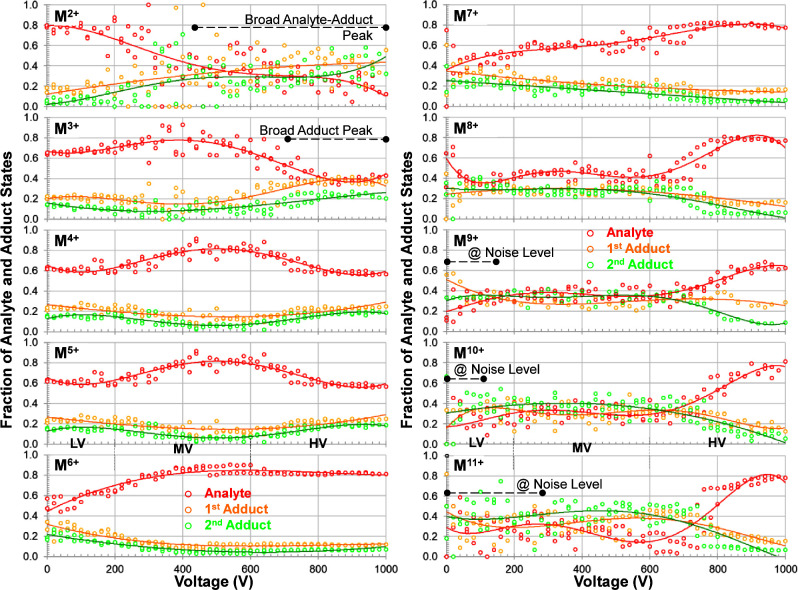
Normalized peak intensities of the first three peaks for the 2+ to 11+ ions as a function of voltage. The panels are labeled according to protein charge state. Peak intensities associated with the protonated (first) peak are represented by the red circles. Yellow and green circles represent the relative abundance of the next two adjacent (higher mass) peaks within the respective ion adduct peak envelopes (Figure S1). For charge states that are not observed above the noise, the voltage range of the noise level is indicated.

To provide greater clarity regarding the related behavior by the ions of the different CSDs, it is useful to plot the relative intensities of the protonated ion versus the first two major adduct ion species centered near ∼+20 and ∼+40 Da (Figure S2). Figure 5 shows this relationship derived for the 2+ to 11+ ions. As mentioned above, the evolution of the protonated peaks with respect to the associated adduct ions for 3+, 4+, and 5+ ions present similar relative intensity patterns. Between +200 to +600 V, the adduct ion features decrease in relative intensity thereby resulting in an increased presence of the protonated peak for these charge states. However, at high voltage (>+600 V), the adduct ion peaks become noticeably larger for these charge states. Comparatively, for the 6+ charge state a greater relative amount of adduct ions is observed even in the absence of any applied voltage. As voltage is increased, the signal of the adduct ion peaks decreases gradually and reaches a plateau at +500 V. At this point, the relative abundance of the protonated peak is ∼70% of the 6+ ions and this remains until the voltage reaches +1000 V. Interestingly, the 7+ to 10+ charge states also display very similar adduct ion production behavior. From the time of their first appearance, a sizable presence of the adduct ions can be observed up to +700 V. Above +700 V, there is a large increase in the relative intensity of the protonated ions relative to the adduct ion peaks.

### Progeny Nanodroplet Makeup for the Different Voltage Regimes

**Figure 6:**
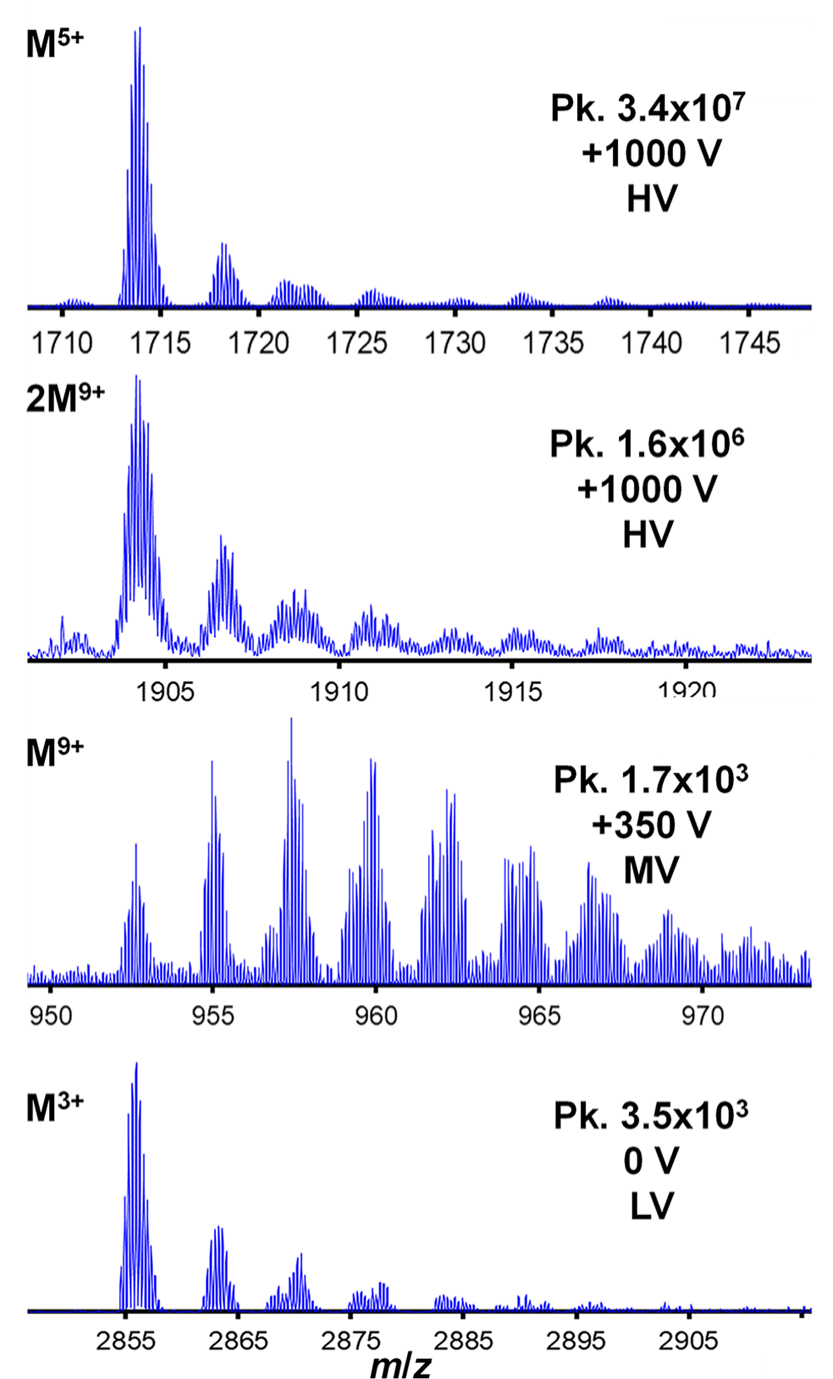
Zoomed-in mass spectra showing adduct ion regions. The top two traces show mass spectra collected at +1000 V (HV). The bottom two traces show mass spectral regions collected at +350 (MV) and 0 V (LV). Protonated ion species are labeled (top left) and voltages are also labeled (bottom right). Spectral regions have been aligned to show overlapping adduct ion species of the form shown in Figure S1.

Here consideration is given as to whether the adduct ion patterns for given charge states can help to confirm similarities in progeny nanodroplet composition under the different voltage conditions. Figure 6 shows zoomed-in regions of mass spectra for different ion species that were collected under HV, MV, and LV conditions. At + 1000 V, 5+ ions, comprising a major feature in the mass spectrum (Figure 4) and assigned to the nESI-type distribution (N2 ensemble Figure 3), show that the protonated ions are the major feature within the adduct ion *m*/*z* range. The single adduct ion is the next most intense data set feature in this region and is nearly four times smaller than the protonated peak. Adduct ions decrease in intensity at higher *m*/*z* values and are observed up to about seven cationic species. Comparatively, the dimer (2M)^9+^ ions exhibit a very similar adduct ion profile at this voltage setting. These data suggest that these ions are ultimately produced from droplets having roughly the same size and composition and via the same ionization pathway. Figure 6 also shows the adduct ion distribution for 9+ protein ions at +350 V. This region represents maximal representation of high charge state ion species (Figure 4). Remarkably, the protonated peaks are not the most intense features of the 9+ protein ions; the first four adduct ions exhibit greater ion intensity values. Additionally, the number of adduct ion series extends to 9 to 10 cations at this voltage.

A final comparison of adduct ion distribution involves the 3+ ions represented as the dominant peak in the mass spectrum at 0 V (Figure 4). In brief, the adduct ion profile of 3+ ions recorded at 0 V is very similar to that of the M^5+^ and 2M^9+^ ions produced at +1000 V (see Figure 6). This may further suggest that the 3+ ions are from a native ensemble of structures just as the nESI-type 5+ and 6+ ions are expected to be. Additionally, it is suggested that the droplet conditions just prior to ion production are similar for these LV and HV voltage regions. This is intriguing considering that prior studies have suggested that although these droplets are initially nearly the same size, their average charge is very different.^15^ Indeed, the average charge of droplets produced at 0 and +1000 V is estimated to be ∼-10,000 *e* and ∼+40,000 *e*, respectively. The question that arises is how are the positive ions produced in a manner that is similar when ultimately arising from these two differing initial droplets? It is probable that droplets from the low-voltage plume undergo aerodynamic breakup^39,65^ to produce positively charged progeny droplets of similar makeup to the progeny droplets from the +1000 V spray. This will need to be further investigated to better understand the preservation of ion structure under zero to low voltage conditions.

### Pathways from Precursor Microdroplets to Progeny Nanodroplets

Ultimately ion production results from multifaceted pathways involving many complex interactions that are nontrivial to model or directly measure. Table 1 lists the properties that have been measured in the lab to better characterize these complex pathways along with some of the properties known from the literature. These are described for three different voltage regimes where the dominant production of the different CSDs and adduct ion species (Figure 4 and Figure 6) are observed. These are again designated the LV (0 to +200 V), MV (+200 to +600 V), and HV (+600 to +1000 V) regimes. To aid the discussion of the pathways droplets experience in the LV, MV, and HV regimes, Figure S3 shows a cartoon representation of the source region from liquid in the cVSSI emitter tip to the precursor ion at the S-lens assembly. In these pathways the liquid is nebulized at the vibrating tip then travels over the ∼1.2 mm tip-to-inlet distance under the influence of the *E*-field (from the DC tip-inlet voltage drop) and the flow-field from the vacuum pulled through the inlet. Once entrained in gas flow of the inlet tube, droplets move down this 60 mm-long heated tube to the low vacuum level (∼2 mbar) of the S-lens assembly.

**Table 1:**
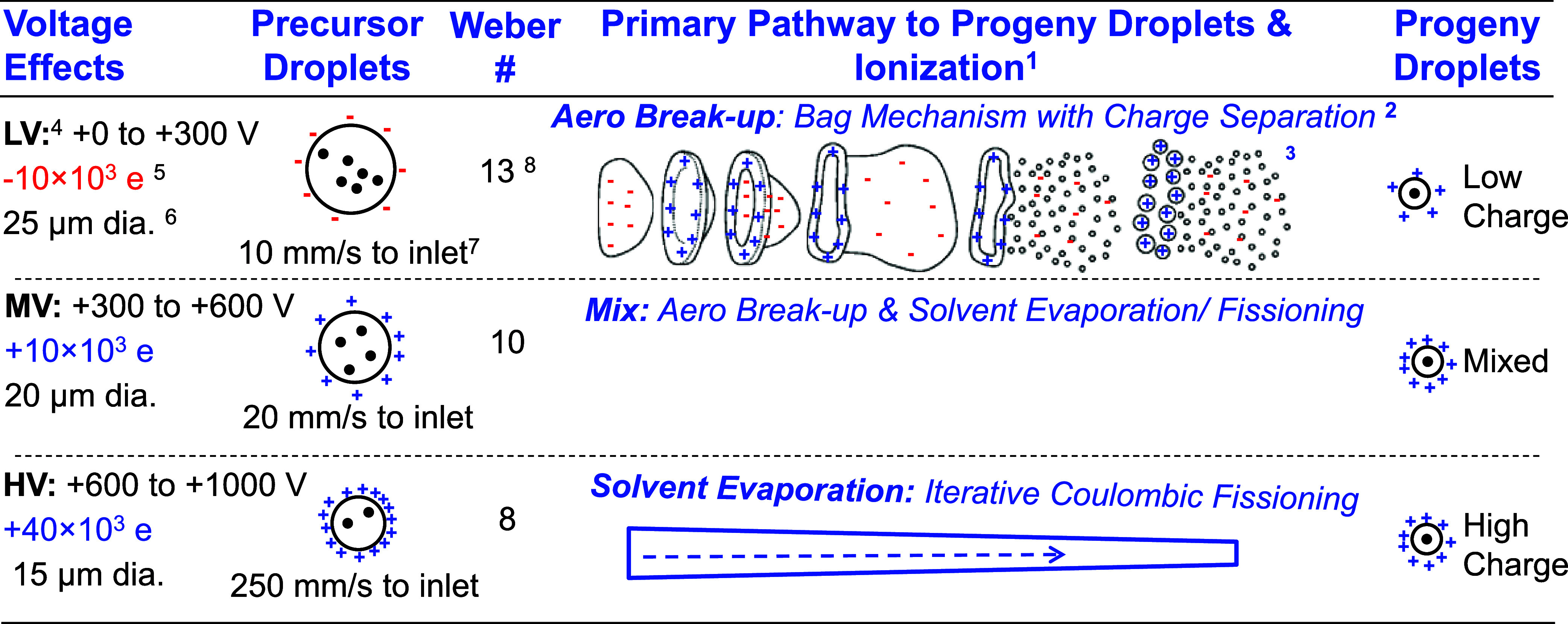
Pathways to Progeny Nanodroplets for Different CVSSI Voltage Regimes

1 Pathway from precursor to progeny droplets used to describe the resulting CSDs (see Figure 5 and Results and Discussion section for details).

2 For information regarding proposed charge separation mechanism see ref (66).

3 Cartoon modified with permission from ref (39).

4 Voltage regimes representing the application of low (LV), medium (MV), and high (HV) voltage to the sample solvent.

5 Charges calculated from measurements of current generated by droplet plume as measured with grounded capillary inlet using a Keysight 34465A.

6 Diameters were measured using diffraction with a laser beam over the full voltage range without the vacuum pulled.

7 Estimated droplet velocities obtained from laser illuminated droplet plumes produced in the different voltage regimes. Velocities estimated with the aid of standard video through the high mag (50× - 500×) microscope

8 Weber # (*We*) is a dimensionless number in fluid mechanics used to characterize an interfacial interaction between a liquid droplet moving relative to a surrounding gas. For *We* > 12 bag aero breakup takes place, whereas for *We <* 10 no breakup takes place. ref (66).

Diffraction, which can directly measure droplet diameters, shows only a modest decrease in size with voltage (Table 1), with typical diameters of 25, 20, and 15 μm in the LV, MV, and HV regimes, respectively. Typical charge on the droplets can be inferred by measuring the current carried by the droplets taking into account the set flow rate and above-measured droplet diameters. These vary markedly over the voltage range used in these experiments. Starting with −ve charged droplets at LV (*Q*∼-10,000 e, based on I = −20 pA) and becoming +ve charged at HV (*Q*∼+40,000 e), with the crossover from −ve to +ve charge typically near +300 V (near the divide between the LV and MV ranges) such that the droplets at MV are mostly + ve (*Q*∼+10,000 e).

The overall pathway includes the interaction regions (Figure S3), where multiple interactions occur. The process sequence involves nebulization, action of the *E*-field especially associated with the high field of the tip, the aerodynamics associated with the flow pattern at the tip inlet, and heating while traveling down the high aspect ratio inlet tube. It is useful to think of these zones acting somewhat independently. The major difference is the droplet charge in these regimes. Moreover, the action of the *E*-field accentuates these differences, so that the velocity of the droplets while the *E*-field dominates (before the vacuum flow field dominates, see Figure S3) is different.

By direct measurement, the droplet velocities are of the order of ∼10 mm/s at LV and well into the MV regime and increase to ∼250 mm/s (estimated by balancing the force of the *E*-field with the viscous force). These different velocities on exiting the *E*-field-dominated region near the tip (Figure S3) and the different droplet diameters will have a pronounced effect on the behavior of the drop in the vacuum flow region. In the vacuum flow region and within the entrance to the inlet, through collisions with gas molecules, the droplet goes from its initial velocity up to ∼150 m/s, orders of magnitude higher than any of the initial velocities. The Weber number (*W*_*e*_)^66,67^ is a dimensionless number in fluid mechanics used to describe the effect of gas flow (fast) on the droplet flow (slow) and is described by , where ρ is the density of the fluid (air), *v* is the relative droplet velocity, *d* is the droplet diameter, and σ is the droplet surface tension. *W*_*e*_ numbers for LV, MV, and HV for the different droplet diameters and velocities are listed in Table 1; they are 13, 10, and 8, respectively. Comparing these *W*_*e*_ numbers to those tabulated^66^ for several experiments we expect *W*_*e*_ > 12 to undergo bag breakup, whereas *W*_*e*_ < 10 to undergo no breakup. Thus, it is likely that the LV large diameter negative droplets (*W*_*e*_ = 13) to undergo bag breakup and **abruptly** become orders of magnitude smaller and the droplets formed from the breakup of the toroidal rim (basal ring) become positive progeny droplets. Whereas, the HV smaller diameter positive droplets (*W*_*e*_ = 8) do not undergo bag breakup but instead gradually undergo solvent evaporation down the length of the ion transfer tube and become positive progeny droplets. The transition from the LV breakup to the HV nonbreakup behavior will take place within the MV range. Figure S3 schematically shows the tip-inlet, ion transfer tube connecting to the S-Lens assembly. In this figure we include a complementary description of the importance of *W*_*e*_. For the 150 m/s molecular flow velocity at the inlet, a Weber diameter, *d*_*We*_, of about 20 μm is determined, where for *d* > *d*_*We*_ bag breakup occurs and for *d* < *d*_*We*_ bag breakup does not take place. Given our measured diameters are 25, 20, and 15 μm for the LV, MV, and HV regimes, respectively, and our *d*_*We*_ is about 20 μm, it can be argued that the LV will experience breakup. In other words, LV involves the slowest and biggest drops acted on by the inlet flow pattern. This implies these drops will be those most likely to be broken apart through aero breakup. In fact, it is likely these large slow drops undergo the well-known bag mechanism,^39,65,67^ which is a mechanism where the large −ve droplets can generate much smaller + ve droplets, which are the progeny droplets of the positively charged ions that are observed. This aero breakup bag mechanism process is expected to continue but decrease in importance into the MV regime. Furthermore, this aero breakup mechanism is not expected to be important in the HV regime where smaller faster drops will more easily be accelerated to the gas velocity at the inlet and their diameters are reduced first through solvent evaporation and then acted on through iterative solvent evaporation and Coulombic fissioning.

Given the complexity of the process from liquid to molecular ions it should not be surprising that varying voltage greatly affects the details of the pathways experienced by the droplets for the different voltages. In summary, for the LV and HV regimes the former are bigger, much slower and of opposite charge than the latter. In the LV regime, the drops likely undergo the bag mechanism near the entrance to the inlet thereby going from the large −ve drops almost immediately to the + ve charged progeny droplets. Whereas in the HV regime the smaller faster already + ve droplets probably undergo the more usual solvent evaporation and Coulombic fissioning route to + ve charged progeny droplets.

### Progeny Droplets as a Selector for Conformer Ensemble Sampling

It is instructive to review the different CSD types depicted in Figure 3 as a means of comparison of progeny droplets and how they relate to the conformer ensemble sampled by the mass spectrometer. The MV regime has larger drops than the HV with much less charge (it includes the crossover from −ve to +ve charge) and slower than the HV. This suggests that for the MV the evaporation aided by Coulombic processes lasts much longer than that in the HV regime corresponding with a greater lifetime under the influence of the heated inlet transfer tube. Therefore, such droplets obtain a greater heat load leading to a shift in ubiquitin conformer ensemble presenting a greater number of partially unfolded states observed as the higher charge states (Figures 3 and 4). For the HV region, the nESI-type CSD dominates (Figure 3). This results from the relatively rapid production of ions. For the LV regime, according to the proposed pathway here, the ions will be formed from positively charged droplets resulting from aero breakup. Based on the comparison of adduct ions shown in Figure 6, these progeny droplets have similar levels of cationic species as those that ultimately produce the nESI-type ions although they must also be of lower net charge.

Because the progeny droplets are similar for the LV and HV CSDs and because these are dominated by ions of low charge, it is suggested that both sample the native conformation ensemble. One difference is noted regarding multimer ions. These ions are not observed in the mass spectra below a voltage of +200 V (LV), as shown in Figure 2. After +600 V (HV) a noticeable increase in multimer ion intensities with a broad plateau from +800 to +1000 V is observed. The stark difference between little multimer intensity in the LV range and maximum intensity in the HV range must be associated with the different pathways to progeny droplets (Table 1) and conformer ensemble selection. Although there is some evidence of a weak interaction to form dimer species,^51^ generally mass spectral multimers of ubiquitin are associated with nonspecific interactions formed by protein coalescence within the drying droplet.^52,53^ The LV pathway involves aero breakup near the start of the mass spectrometer inlet (Figure S3) wherein the large initial droplet abruptly breaks into droplets orders of magnitude smaller. So, it can be argued that the likelihood of multimer formation is decreased due to protein partitioning in the aero breakup. This extends into the MV region; however, droplets here may also select for decreased multimer formation due to the increased droplet heating (see above). In contrast, the HV pathway initially involves solvent evaporation before Coulombic fissioning and thus the partition process experienced here could result in greater protein number on average within progeny droplets. As mentioned above, multimer ions having up to nine proteins (e.g., [9M+20H]^20+^) form.

The possibility of protein partitioning upon nanodroplet production during aerodynamic breakup is intriguing. As further support for ion partitioning, consider Figure 6 where the [M+3H]^3+^ ions exhibit relatively little adduct ion formation. This would argue that small species are partitioned upon droplet aero breakup along with the much larger proteins. This may effectively dilute charge carriers responsible for adduct ion formation in much the same way that submicrometer nESI tips function as demonstrated in the notable work of the Williams research group.^68^ Overall, the decreased adduct ion and multimer ion formation at low voltages can both be explained by such a partitioning mechanism.

### Other Forms of Droplet Heating in the MV Regime?

Considering the complexity of ion formation discussed thus far, it is useful to consider another potential contributor to droplet heating in the MV regime. The movement of a relatively small charge over very short distances could result in a sizable alternating *E*-field. Figure S2 shows the estimated RF field created by an oscillating (95 kHz) cVSSI emitter tip to which +350 V has been applied. Notably, using a value of 20 μm oscillating amplitude for the tip, the RF field is greatest just after the tip (1 to 30 μm). Here, a peak-to-peak *E*-field of 1,400 V/mm is obtained. This corresponds with a change in the reduced electric field ratio (*E*/*N*, where *E* is the *E*-field strength and *N* is the neutral number density) of ∼50 Townsend (Td) in the region just after the emitter tip (Figure S1). Figure S2 also shows that this oscillating field dissipates rapidly along the axis of the ion generation pathway. By a distance of 150 μm from the tip, the *E*/*N* changes by just 0.6 Td. Based on these numbers it is proposed that, for these experiments, RF heating is not the major factor contributing to droplet heat loading in the MV regime. Indeed, changing the inlet transfer tube temperature to 200 °C (from 275 °C) delays evidence for ubiquitin unfolding by ∼+100 V (manuscript in preparation). Therefore, it is here suggested that droplet residence times under active heating from the transfer tube are the largest factor affecting protein unfolding. That said, the *E-*field simulations suggest that at higher vibration amplitudes, RF heating should be examined more carefully as a potential source of droplet heating.

## Conclusions

For spray-based ionization sources, the exact influence of the droplet charge on the ionization process of proteins is not entirely understood. The decoupling of droplet production and charging in cVSSI provides the opportunity to select the overall charge of the droplets and monitor the types of ions produced. Here the CSDs of ubiquitin have been monitored across the 0 to +1000 V range. According to CSD transitions, notable changes in protein ion conformation ensembles sampled by the mass spectrometer are observed. In the LV range, the presence of low charge states (2+, 3+, and 4+) is suggestive of native conformer ensemble selection during the ionization process. In the MV range, the high charge states (7+ to 12+) of ubiquitin are observed indicating a shift in the conformer ensemble to partially unfolded species. In the HV range, the CSD is dominated by nESI-type ions (5+ and 6+) suggesting again a sampling of the native conformer ensemble.

Comparisons of droplet characteristics can explain the origin of the conformer ensemble sampled by the mass spectrometer. The low charge, slow moving droplets of the LV region undergo aero-breakup to achieve charge separation. This results in progeny droplets that produce very low charge states arising from the native conformer ensemble absent the multimers due to protein partitioning in the breakup. In the MV region, the relatively slow moving and large droplets experience greater interaction with the heated capillary prior to ion production ultimately resulting in a shift in the conformer ensemble sampled by the mass spectrometer. For the HV regime, the greater Coulombic fissioning and Coulombically aided desolvation results in earlier ion production and thus less droplet heating. This process samples the native conformer ensemble as is typical of nESI to include the multimer ions.

The data presented above are the first to begin characterizing ionization processes for the new ionization technique known as cVSSI. Because it decouples droplet production from Coulombically driven process associated with ESI, cVSSI offers the unique opportunity to study the different factors (droplet size and charge and transfer tube inlet temperature) affecting ion production. In the future, more exhaustive studies will be performed to characterize the effect of each factor. Additionally, the studies will focus on examining the effects for a wide variety of proteins exhibiting different stabilities in solution. Because of the tremendous enhancement in ion production for negatively charged ions from aqueous solutions by cVSSI,^43,69^ these studies may be especially beneficial for native MS experiments of important biopolymers such as DNA and RNA and their complexes. Finally, because the approach is tunable (Figures 4), it is possible that voltage profiles can be generated to rapidly reveal differences in protein or biopolymer stabilities and/or changes in such upon ligand binding.

## Supporting Information Available

Plots of two mass spectral regions, schematic diagram of the droplet production, and plot of hypothetical electric field in the source region (PDF)
