# Supplementary material for: Accessing Different Protein Conformer Ensembles with Tunable Capillary Vibrating Sharp-Edge Spray Ionization
Source: J Phys Chem B. 2025 Jan 29;129(5):1626–39. doi: 10.1021/acs.jpcb.4c04842 (PMC11808649; doi:10.1021/acs.jpcb.4c04842)
Supplement: Supplementary file 1 — jp4c04842_si_001.pdf [file jp4c04842_si_001.pdf]

Accessing Different Protein Conformer Ensembles with Tunable Capillary Vibrating Sharp-edge Spray Ionization

Daud Sharif,[1]□ Vikum K. Dewasurendra,[2]□ Mst Nigar Sultana,[1] Sultan Mahmud,[1] Chandrima Banerjee,[1] Mohammad Rahman,[1] Peng Li,[1] David E. Clemmer,[3] Matthew B. Johnson,[2*] Stephen J. Valentine[1*]

[1]Department of Chemistry, West Virginia University, Morgantown, WV 26506, United States
[2]Department of Physics, West Virginia University, Morgantown, WV 26506, United States
[3]Department of Chemistry, Indiana University Bloomington, Bloomington, IN 47405, United States

Supporting Information

Figure S1. Zoomed-in mass spectral regions of the 3+ to 11+ ubiquitin ions S2

Figure S2. Zoomed-in mass spectral region of $[M+6H]^{6+}$ ubiquitin ions S3

Figure S3. Schematic representation of the droplet to ion production region of the experiment S4

Figure S4. Plot of the hypothetical electric field produced by cVSSI at +350 V S5

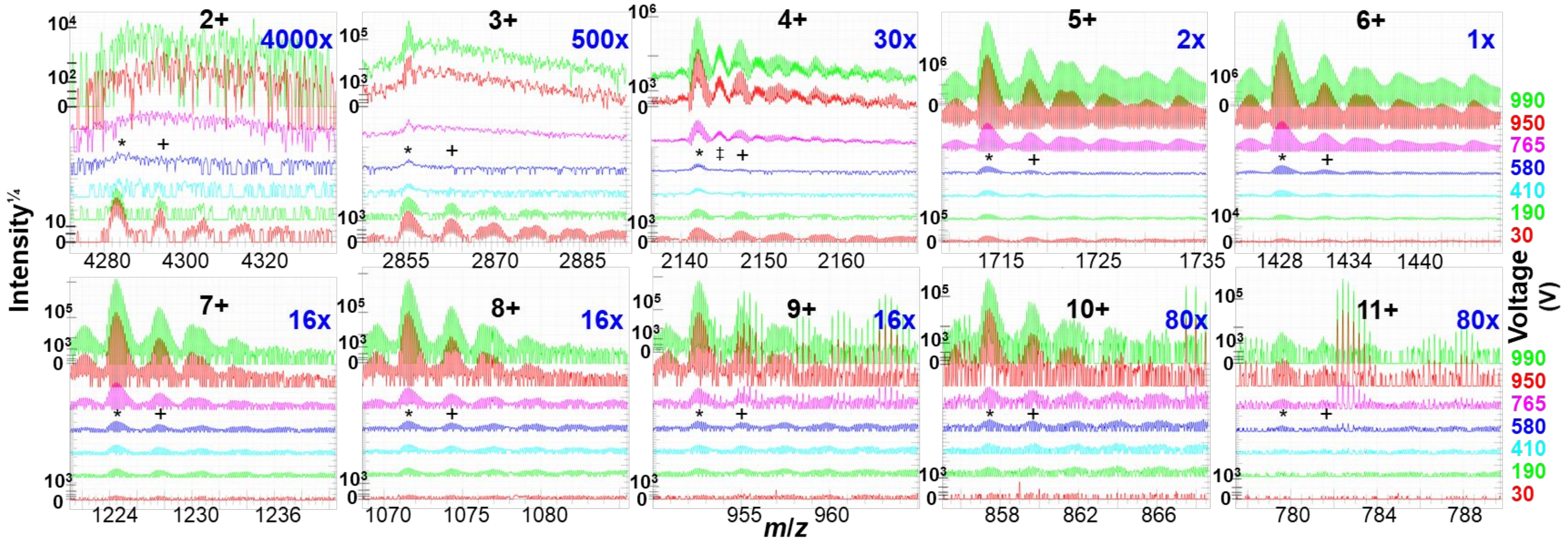

Figure S1. The peak envelopes of different charge states showing the distribution of adduct ions. Each panel shows stacked mass spectral regions as a function of applied voltage (from 0 to +1000 V). From bottom to top, envelopes are shown at different voltage settings. The panels are labeled as M^{z+} where M indicates monomer ubiquitin and z represents the charge. Peak intensities are shown on a $y^{1/4}$ scale to aid visualization of adduct ion species. In each stack, the relative intensity amplitude is the same. Between stacks, to best display the details of each charge state, the intensity has been multiplied by the factor shown for each stack (top right). The m/z scale for each stack has been scaled so the isotope (and adduct) peak spacing are the same for each stack. This same spacing allows the characteristic shapes of the adduct peaks to be compared between charge states for different voltages. The * symbols indicate the position of the protonated peak (zero adduct) and the + symbols indicate the position of the first adduct. The ‡ symbol indicates a multimer peak associated with $[2M]^{8+}$.

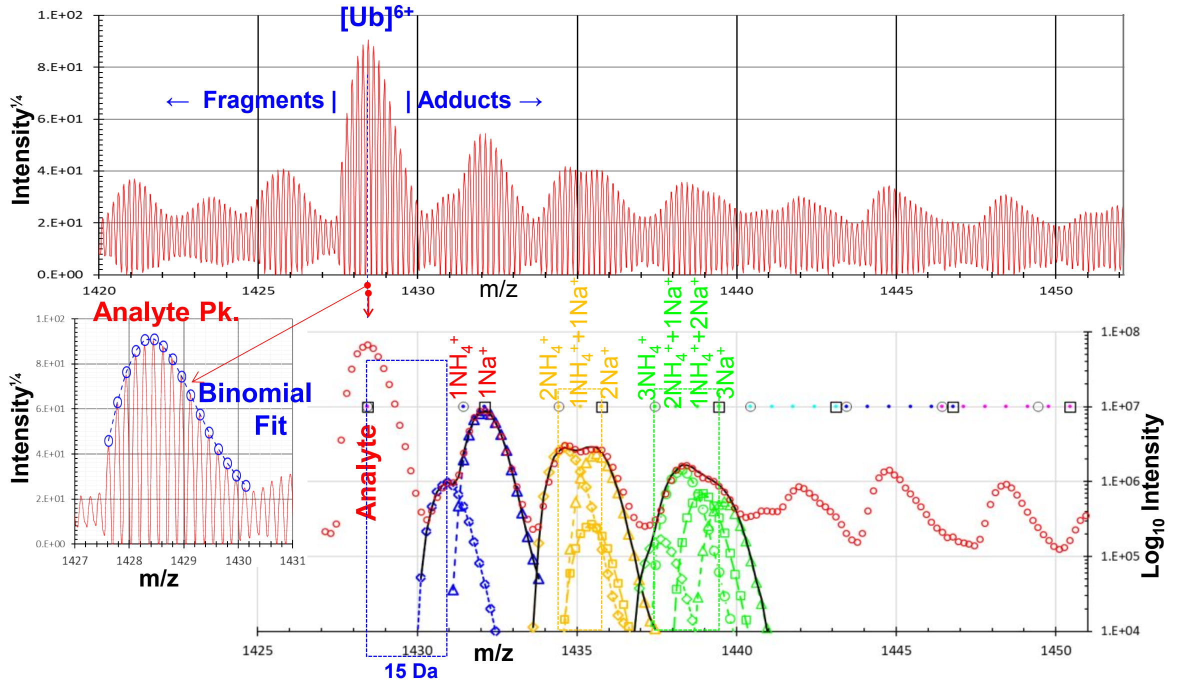

Figure S2. Experimental and theoretical adduct ions for 6+ ubiquitin ions. The top trace shows a zoomed-in region of a mass spectrum collected with the application of +1000 V. The 6+ ions are labeled. The inset (left) shows an expanded region for the $[M+6H]^{6+}$ ion feature. Also shown is the binomial distribution ($^{13}C/^{12}C$) fit for these ions. The bottom trace shows the experimental isotopologue peak apexes (red circles) as well as the predicted isotopologue apexes for single, double, and triple adduct ion species represented by the blue, yellow, and green symbols, respectively. Proposed adduct ion series are shown above with various combinations of NH_4^+ and Na^+ adducts. The black traces reveal the best fits for the hypothetical adduct ions. Ion intensities are presented on a $y^{1/4}$ scale to emphasize adduct contributions. The peak fitting the shoulder centered at the position of +15 Da above the analyte is possibly due to overlapping peaks at +14 and +16 Da possibly caused by the methylation of arginine (+14 Da) or the oxidation of methionine (+16 Da).

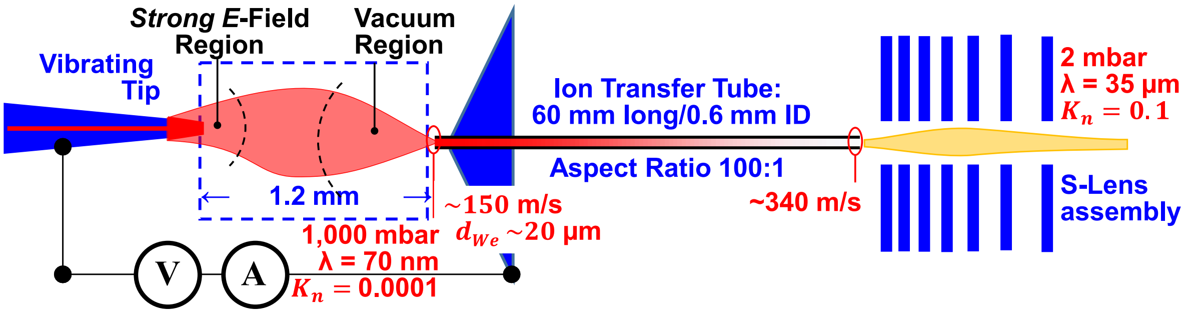

Figure S3. Schematic representation of the droplet to ion production region of the experiment. Shown are the vibrating tip, the plume visualization, capillary inlet, S-lens assembly regions. Regions and sub-regions are labeled. Physical characteristics of each region such as mean free path (λ), pressure, and Knudsen number (Kn) are provided. Physical dimensions of the capillary inlet and tip to inlet distance are provided. Finally, estimated droplet velocities in different regions and Weber diameter (d_{We}) at the inlet are provided.

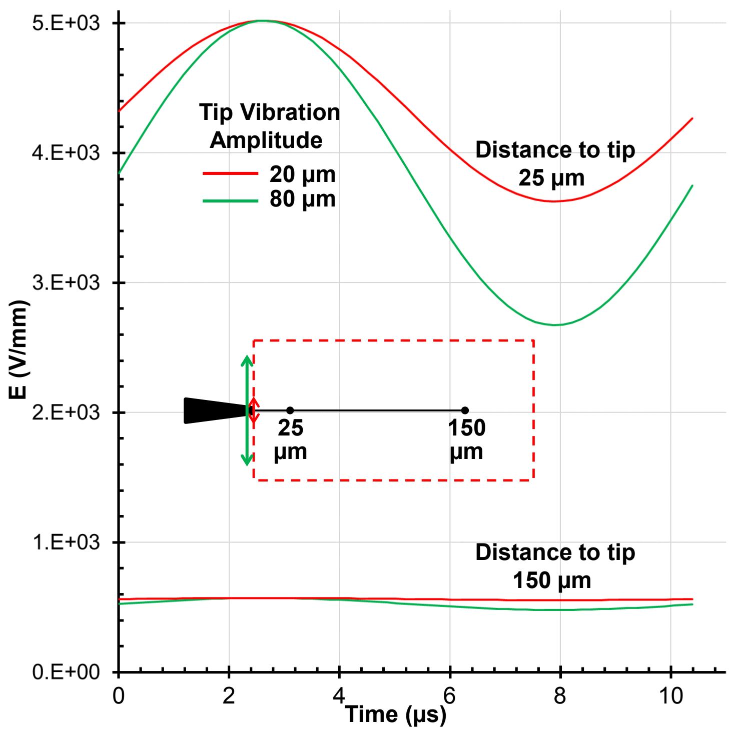

Figure S4. Plot of the hypothetical electric field produced by cVSSI at +350 V. The position of field determination are provided above each set of traces and represent distance from the cVSSI emitter tip to the capillary inlet of the mass spectrometer. The legend provides the color code for cVSSI emitter tip amplitude. Electric field traces represent first approximations using the online field generator at https://icphysweb.z13.web.core.windows.net/simulation.html.
